# Southern African HIV Clinicians Society 2022 guideline for the management of sexually transmitted infections: Moving towards best practice

**DOI:** 10.4102/sajhivmed.v23i1.1450

**Published:** 2022-09-27

**Authors:** Remco P.H. Peters, Nigel Garrett, Nomathemba Chandiwana, Ranmini Kularatne, Adrian J. Brink, Karen Cohen, Katherine Gill, Thato Chidarikire, Camilla Wattrus, Jeremy S. Nel, Mahomed Y.S. Moosa, Linda-Gail Bekker

**Affiliations:** 1 Research Unit, Foundation for Professional Development, East London, South Africa; 2 Department of Medical Microbiology, University of Pretoria, Pretoria, South Africa; 3 Division of Medical Microbiology, Faculty of Health Sciences, University of Cape Town, Cape Town, South Africa; 4 Centre for the AIDS Programme of Research in South Africa (CAPRISA), University of KwaZulu-Natal, Durban, South Africa; 5 Department of Public Health Medicine, School of Nursing and Public Health, University of KwaZulu-Natal, Durban, South Africa; 6 Ezintsha, Faculty of Health Science, University of the Witwatersrand, Johannesburg, South Africa; 7 Department of Clinical Microbiology and Infectious Diseases, Faculty of Health Sciences, University of the Witwatersrand, Johannesburg, South Africa; 8 Department of Medicine, Division of Clinical Pharmacology, University of Cape Town, Cape Town, South Africa; 9 Desmond Tutu HIV Centre, University of Cape Town, Cape Town, South Africa; 10 National Department of Health, Pretoria, South Africa; 11 Southern African HIV Clinicians Society (SAHCS), Johannesburg, South Africa; 12 Helen Joseph Hospital, University of the Witwatersrand, Johannesburg, South Africa; 13 Department of Infectious Disease, Division of Internal Medicine, Nelson R. Mandela School of Medicine, University of KwaZulu-Natal, Durban, South Africa

## Contents

1. Introduction
  TABLE 1. Microbial aetiology of sexually transmitted infection syndromes
2. Screening for STIs
  2.1. Provider-initiated STI-symptom screening
  2.2. STI screening using diagnostic tests
  TABLE 2. Recommended diagnostic screening frequency for specific sexually active population groups
3. Clinical management of the symptomatic patient
  3.1. Management of male urethral discharge syndrome (MUDS)
  TABLE 3. Recommended first-line antimicrobial treatment regimens for STI syndromic management
  3.2. Management of vaginal discharge syndrome (VDS)
  3.3. Genital ulcer disease (GUD)
4. Engaging sex partner/s in care
5. Clinical management of patients with recurrent or persistent symptoms
  TABLE 4. Reasons for persistent or recurrent STI episodes
6. Diagnostic testing for sexually transmitted infections
  6.1. *Chlamydia trachomatis, Neisseria gonorrhoeae* and *Trichomonas vaginalis*
  TABLES 5a and 5b. Recommended diagnostic tests for management of sexually transmitted infection-associated symptoms – Genital discharge syndromes
  6.2. *Mycoplasma genitalium*
  6.3. HSV-1 and HSV-2
  6.4. *Treponema pallidum*
7. Pathogen-directed treatment of specific sexually transmitted infections
  7.1. *Chlamydia trachomatis*
  TABLE 6. Recommended antimicrobial drugs for targeted treatment of uncomplicated sexually transmitted infections
  7.2. *Neisseria gonorrhoeae*
  7.3. *Trichomonas vaginalis*
  7.4. *Mycoplasma genitalium*
  7.5. HSV-1 and HSV-2
  7.6. *Treponema pallidum*
8. Conclusion
  Acknowledgments
    Competing interests
    Authors’ contributions
    Ethical considerations
    Funding information
    Data availability
    Disclaimer
  References

## 1. Introduction

Sexually transmitted infections (STIs) are among the most common acute conditions worldwide with sub-Saharan Africa ranking among the regions with the highest burdens globally.^1^ Adolescent girls and young women (AGYW), people living with HIV (PLHIV), pregnant women, and key and vulnerable populations are disproportionally affected by STIs. The social determinants of health, gender inequality, and STI-associated stigma and discrimination (at both the community and facility level) are important contributors to the sustained high burden of infection.

Some STIs cause urogenital infections including urethritis, cervicitis, vaginitis and genital ulceration, and may also infect the rectum and pharynx. Other STIs may cause serious short-term and long-term complications (e.g. pelvic inflammatory disease, arthritis, encephalitis), increase the risk of ectopic pregnancy and tubal-related infertility, and are associated with stillbirth and other adverse pregnancy outcomes.^2^ Furthermore, susceptibility to acquiring HIV as well as HIV infectiousness may be increased depending on which STI is involved.^3^ Lastly, the emergence of antibiotic-resistant gonorrhoea and consequent limited therapeutic options has become a major public health concern, impacting STI management and control activities.^4^

The World Health Organization’s (WHO) Global Health Sector strategy 2022–2030 details the vision, goals and actions to ending the STI epidemic.^5^ Strengthening STI case management is one of the key priorities of this strategy. Effective people-centred STI case management is context-dependent and determined by available resources.^6^ In settings with limited resources, despite its shortcomings, syndromic case management using flow charts is the standard of care. Male urethral discharge syndrome (MUDS), vaginal discharge syndrome (VDS) and genital ulcer disease (GUD) are the main STI-associated conditions in this approach, and each of these syndromes has a diverse microbial aetiology (Table 1).^6,7,8,9,10,11,12,13,14^ Where resources are available, diagnostic tests for specific pathogens, combined with directed treatment, can be used to optimise STI screening and case management.

**TABLE 1:** Microbial aetiology of sexually transmitted infection syndromes.

Syndrome	Common aetiology (> 10% of cases)	Less common aetiology (< 10% of cases)
Male urethral discharge syndrome	*Neisseria gonorrhoeae Chlamydia trachomatis*	*Mycoplasma genitalium Trichomonas vaginalis* Other aetiology
Vaginal discharge syndrome	*Neisseria gonorrhoeae Chlamydia trachomatis Trichomonas vaginalis* Bacterial vaginosis Candidiasis	*Mycoplasma genitalium* Non-infectious aetiology
Genital ulcer syndrome	HSV-1 and HSV-2 *Treponema pallidum*	*Chlamydia trachomatis* biovar Lymphogranuloma venereum *Haemophilus ducreyi Klebsiella granulomatis*

HSV, herpes simplex virus.

This guideline provides basic recommendations to aid appropriate STI case management in the Southern African primary care setting. Acknowledging that available resources vary between settings, and since this guideline applies to both private and public health sectors, recommendations may on occasion be aspirational in the public sector but feasible within the private sector. Further detailed guidance for the management of STIs can be found in the 2021 WHO and 2021 Centers for Disease Control and Prevention (CDC) guidelines.^6,15^

## 2. Screening for sexually transmitted infections

Screening for STIs is essential to reduce the burden of infection, morbidity, and associated health complications within the population. This should be done by asking about STI-associated symptoms followed by a clinical work-up if indicated or, in some instances, by diagnostic screening of asymptomatic individuals.

As part of a comprehensive sexual health assessment, in addition to taking history and conducting physical examination, HIV testing, testing for hepatitis B virus (HBV), hepatitis C virus (HCV), syphilis, and cervical cancer screening (oncogenic human papillomavirus [HPV] DNA testing or Pap smear) should be done in line with local guidelines and resource availability. HIV pre-exposure prophylaxis (PrEP) may be offered in line with the local guidelines.^16,17^

### 2.1. Provider-initiated sexually transmitted infection-symptom screening

Provider-initiated STI-symptom screening should be the standard of care in the primary care context, especially during provision of antenatal care, sexual and reproductive healthcare, HIV testing, HIV PrEP and antiretroviral therapy (ART) services. Although there is no documented evidence, provider-initiated symptom screening is likely to result in the identification and treatment of a larger number of symptomatic cases when compared to reliance on patient self-reporting. Some patients may be unaware of their symptoms, including women who may not always realise or report a new or changed vaginal discharge.^18^ In addition, personal perceptions, beliefs and stigma related to sexual health may contribute to creating a barrier to active self-reporting of such symptoms.^19,20,21^ Creating an enabling and supportive environment is vital to overcoming such barriers, including reducing the reluctance of healthcare workers to proactively ask about STI-associated symptoms.^22^ Therefore, regular provider-initiated STI-symptom screening is recommended as a feasible intervention that requires little time, effort and cost.

### 2.2. Sexually transmitted infection screening using diagnostic tests

Screening for STIs using diagnostic tests followed by specific pathogen-directed treatment shortens the duration of infection, reduces ongoing transmission, may reduce complications, and ultimately may result in a lower STI prevalence in populations.^15,23,24,25^ In addition, diagnostic screening of asymptomatic individuals (with the associated risk of complications and transmission to sexual partners) can identify those patients that would remain untreated using the symptom-based screening approach. In fact, few STIs are symptomatic. It is estimated that only 11% – 33% of *Chlamydia trachomatis* infections in men, and 6% – 17% in women, become symptomatic; estimates for symptomatic *Neisseria gonorrhoea*e infection are 45% – 85% in men and 14% – 35% in women.^26,27^ Another benefit of diagnostic STI screening over symptom-based screening is improved antimicrobial stewardship and optimisation of partner management.

Recommendations for diagnostic testing of asymptomatic individuals should take into account the STI prevalence, sexual behaviour, disease severity and sequelae, health impact and cost.^15,21^ Based on these criteria, recommendations have been made for diagnostic screening in various population groups, and screening frequency detailed based on the individual’s profile (Table 2).

**TABLE 2:** Recommended diagnostic screening frequency for specific sexually active population groups.

Population group	CT NAAT	NG NAAT	TV NAAT	TP Serology	Suggested screening frequency
Adolescent girls and young women	x	x	-	x	At least annually based on risk assessment†
Commercial sex workers	x	x	x	x	3–6 monthly
Individuals at increased risk†	x	x	-	x	At least annually
Men who have sex with men	x	x	-	x	At least annually, at least 6 monthly if increased risk†
People living with HIV	x	x	-	x	At least annually, and especially on entry to care
Pregnant women	x	x	x	x	At first antenatal visit, and repeat in third trimester (32 weeks)
PrEP users	x	x	-	x	At PrEP initiation then at least annually
Transgender and gender diverse	x	x	-	x	At least annually, more frequently based on risk assessment†

CT, *Chlamydia trachomatis*; NAAT, nucleic acid amplification test; NG, *Neisseria gonorrhoeae*; TV, *Trichomonas vaginalis*; TP, *Treponema pallidum*; PrEP, pre-exposure prophylaxis.

† , Individuals are considered at increased risk if: multiple sex partners, engaging in transactional sex, sex under influence of drugs, sexually transmitted infection diagnosis in the last year.

In the case of diagnostic testing, detection of *C. trachomatis, N. gonorrhoeae* and *Trichomonas vaginalis* should be done using a nucleic acid amplification test (NAAT) using first-void urine in men and, in women, a self-collected or healthcare-worker-collected vulvovaginal or endocervical swab. In some specific situations, collection of first-void urine may provide an alternative option to a vaginal swab; however, a vaginal swab is the preferred specimen as the yield is higher than with urine in women.^6^ Anorectal and pharyngeal specimens for *N. gonorrhoeae* should also be considered in men who have sex with men (MSM), transgender and gender diverse people (TGD), and commercial sex workers (CSW) dependent on individual sexual practices.^21^ Serological testing for syphilis should be done using a treponemal test, followed by a nontreponemal test if the treponemal test result was positive. Rapid diagnostic tests (RDTs) for syphilis (alone or in combination with HIV) have recently become available and provide a good point-of-care screening test option.^28^

Screening of asymptomatic individuals for *Mycoplasma genitalium* is not recommended due to its unclear pathogenicity and concerns of rising antimicrobial resistance (AMR) associated with treatment.^15,29^ Serological screening for herpes simplex virus (HSV) is discouraged as a positive screening result has no clinical implication because of a lack of therapeutic options to eradicate latent HSV alongside the high seroprevalence in Southern Africa.^15,30^

## 3. Clinical management of the symptomatic patient

A comprehensive clinical work-up of a patient with STI-associated symptoms should include a sexual, urological, medical (including medication history, vaccination, and medication allergies) and, for women, gynaecological history (including contraception and pregnancy). Physical examination of genital, oral and anal areas should be conducted, and testing for HIV and syphilis carried out. Women should be assessed for symptoms and signs suggestive of pelvic inflammatory disease (PID), including an examination for lower abdominal tenderness. Cervical cancer screening should be discussed and, if indicated, HPV DNA testing (as the preferred option) or a Pap smear should be conducted in line with the local available resources, guidelines and policy.^31^

In the absence of diagnostic tests, syndromic treatment (i.e. empirical antimicrobial treatment that covers the most likely aetiology of the syndrome that the patient presents with) should be provided and patients instructed to return for further management if there is not resolution of symptoms. Syndromic management algorithms and treatment regimens differ between countries based on the local epidemiology and access to medications; however, general best practice recommendations are provided. Management of STIs with systemic presentations (e.g. disseminated gonococcal infection), epididymo-orchitis and PID) are beyond the scope of this guideline. Anorectal discharge has a broad aetiology including presence of an STI. Clinical assessment and work-up of anorectal discharge and proctitis are not included in this guideline.

### 3.1. Management of male urethral discharge syndrome

Male urethral discharge syndrome should be confirmed with a physical examination. A urethral discharge may be seen directly but, if not, the penis should be milked. Examination of the epididymis and testes is also recommended. Most MUDS cases are caused by *C. trachomatis* and/or *N. gonorrhoeae* infection. Therefore, the recommended regimen for uncomplicated MUDS is a combination of azithromycin, to treat *C. trachomatis,* with ceftriaxone to treat *N. gonorrhoeae* (Table 3). In syndromic management settings, in the absence of diagnostic testing, azithromycin is considered the preferred choice to cover treatment for *C. trachomatis* infection in the syndromic regimen. Although doxycycline has a slightly higher efficacy than azithromycin for urogenital infection in men (lower rate of microbiological failure but not clinical failure) but not women, there are no data that support higher effectiveness of a course of doxycycline for *C. trachomatis* treatment in syndromic management settings. In the absence of diagnostics, a single dose of azithromycin is considered to outweigh the benefit of the higher efficacy of a course of doxycycline due to its better tolerability and possible adherence issues with a course of treatment.

**TABLE 3:** Recommended first-line antimicrobial treatment regimens for sexually transmitted infection syndromic management.

Syndrome	First-line option	Effective substitutes
Male urethral discharge syndrome	Azithromycin 1 g, orally, single dose† *PLUS* Ceftriaxone 500 mg, intramuscularly, single dose	Azithromycin 1 g, orally, single dose† *PLUS* Cefixime 800 mg, orally, single dose
Vaginal discharge syndrome	Azithromycin 1 g, orally, single dose *PLUS* Ceftriaxone 500 mg, intramuscularly, single dose *PLUS* Metronidazole 400 mg or 500 mg, orally, twice daily for 7 days‡,§ *OR* Metronidazole 2 g, orally, single dose‡,§	Azithromycin 1 g, orally, single dose *PLUS* Cefixime 800 mg, orally, single dose *PLUS* Metronidazole 400 mg or 500 mg, orally, twice daily for 7 days‡,§ *OR* Metronidazole 2 g, orally, single dose‡,§
Symptoms and signs suggestive of candidiasis	Miconazole vaginal pessaries, 200 mg, inserted at night for 3 nights *OR* Clotrimazole vaginal tablet, 500 mg, inserted at night single dose	Fluconazole 150 mg or 200 mg, orally, single dose§ *OR* Nystatin, 200–300-unit vaginal tablet, inserted at night for 7 nights
Genital ulcer disease	**First episode**†† Acyclovir 400 mg, orally, three times a day for up to 10 days *PLUS* Benzathine benzylpenicillin 2.4 MU, intramuscularly single dose **Recurrent episode**†† Acyclovir 400 mg, orally, three times a day for 5 days *PLUS* Benzathine benzylpenicillin 2.4 MU, intramuscularly single dose	**First episode**†† Acyclovir 400 mg, orally, three times a day for up to 10 days *PLUS* Doxycycline 100 mg, orally, twice daily for 14 days¶ **Recurrent episode**†† Acyclovir 400 mg, orally, three times a day for 5 days *PLUS* Doxycycline 100 mg, orally, twice daily for 14 days¶

† , In the absence of diagnostics, azithromycin is preferred over doxycycline in syndromic management of symptomatic individuals.

‡ , Avoid metronidazole in first trimester of pregnancy.

§ , In case of good adherence, a course of metronidazole is preferred over single-dose treatment as it has higher efficacy for *T. vaginalis* than single-dose metronidazole and has the added benefit of treating concurrent bacterial vaginosis.

¶ , Doxycycline for men and non-pregnant/non-breastfeeding women; avoid in pregnancy.

†† , Treatment may be restricted to acyclovir if the patient reports no sexual contact in the previous 3 months, and in individuals with recurrent vesicular ulcers in same site and with a recent history (< 3 months) of syphilis treatment (RPR monitoring to be done).

Ciprofloxacin should be avoided in a syndromic regimen due to the high rates of AMR of *N. gonorrhoeae* globally.^4,32^ Treatment for *T. vaginalis* is usually not included because the prevalence in men is too low to justify coverage of this pathogen in first-line syndromic treatment regimens. Patients with MUDS should be instructed to return if there is no resolution of symptoms if that is the case and ideally, resources permitting, collection of a urethral discharge smear for light microscopy and/or first-catch urine or urethral swab for STI diagnostic testing undertaken. Pathogen-directed same-day treatment and partner management should then be provided (see the pathogen-directed treatment section).

### 3.2. Management of vaginal discharge syndrome

The aetiology of VDS is more diverse than that of MUDS. In addition to *C. trachomatis, N. gonorrhoeae* and *T. vaginalis* infection, bacterial vaginosis (BV) and vulvo-vaginal candidiasis (VVC) are important conditions to consider. Occurrence of BV and VVC is unrelated to sexual activity and may present in combination with an STI or as stand-alone conditions.

Given the high burden of STIs in Southern Africa, the syndromic treatment regimen of VDS should cover *C. trachomatis, N. gonorrhoeae* and *T. vaginalis* infection in sexually active women. The recommended empirical regimen is azithromycin, ceftriaxone, and metronidazole (Table 3). A 7-day course of metronidazole is preferred over single-dose treatment as it has a higher efficacy for the treatment of *T. vaginalis* and, if present, the added benefit of treating concurrent BV.^15,33^ However, single-dose metronidazole may be used in certain populations and settings based on the benefits of same-day and observed therapy, and medication availability. Ideally, patients with VDS should be reviewed after one week to confirm resolution of their symptoms.

Most cases of VVC are caused by *Candida albicans* which is generally susceptible to imidazole, polyene and triazole medications. Treatment for VVC may be provided to women with VDS, either in combination with syndromic treatment or as stand-alone management, based on sexual history and examination findings. Typical symptoms of *C. albicans* include vulvo-vaginal pruritus, a burning sensation of the vulva, and vaginal pain or irritation. In addition, dyspareunia and dysuria may be reported. If a vaginal discharge is present with VVC, it is usually thick, curdy and white or cream in colour. Intravaginal pessaries or tablets are recommended first-line treatment (Table 3).

To support syndromic management, if resources permit, a vaginal discharge smear for light microscopy or a self-collected or healthcare-worker-collected vulvovaginal swab may be taken for diagnostic testing for *C. trachomatis, N. gonorrhoeae and T. vaginalis*. These tests are useful in providing same-day pathogen-directed treatment, reducing the use of unnecessary antimicrobials and risk of AMR, and help inform partner management.

### 3.3. Genital ulcer disease

The manifestation of GUD is diverse and the characteristics of the ulcer (e.g. presence or absence of pain, shape of edges, multiplicity) are of poor diagnostic value in determining aetiology, particularly in PLHIV.^34^ Attempting to clinically diagnose the aetiology of GUD using ulcer characteristics is not recommended and should not be used to inform treatment decisions. HSV-1 and HSV-1 are the most common causes of genital ulcers followed by *Treponema pallidum*, the causative agent of syphilis. Lymphogranuloma venereum (LGV) caused by *C. trachomatis* biovars L1–L3, chancroid (*Haemophilus ducreyi*), and donovanosis (*Klebsiella granulomatis*) have become uncommon in the last decade.^14,35,36^

First-line syndromic regimen for GUD should include medications to treat HSV and syphilis (Table 3). Acyclovir is the medication of choice for HSV while benzathine benzylpenicillin, also called benzathine penicillin G (BPG), is the cornerstone of syphilis treatment. Treatment for chancroid should only be considered in a setting where cases are reported or emerging, while LGV treatment should be based on an aetiological diagnosis. Limiting empirical treatment of GUD to acyclovir (i.e. omitting BPG) may be considered in individuals with a recent history (< 3 months) of adequate syphilis treatment, those who present with recurrent ulcers in the same site, and in individuals reporting no sexual contact in the previous three months. Patients with GUD should be reassessed for symptoms after one week of treatment. Blood for rapid plasma reagin (RPR) titre should be collected at initial presentation. Serological monitoring of RPR for response to treatment may be considered 3–6 months after the completion of treatment and, if indicated, every 3–6 months thereafter. A four-fold (two dilution step) decrease in the RPR titre can be expected following 12-months of treatment in HIV-uninfected people, and following 24 months treatment in those that are HIV-infected.^15^ Patients presenting with GUD and/or a diagnosis of syphilis should be assessed for neurological symptoms and, if present, referred for treatment.

Resources permitting, a swab of the genital lesion may be collected for NAAT testing of HSV and *T. pallidum*. An HSV NAAT assay is the most sensitive test for HSV (sensitivity > 90%), and is considered highly specific.^15,37^ Discontinuation of acyclovir may be considered in the case of a negative HSV NAAT. Molecular tests for syphilis have a lower sensitivity (80% – 90%) and specificity. Therefore, these tests should not be used to exclude syphilis but can facilitate early diagnosis in the case of a positive result.^38,39^

Herpes simplex virus serology should be avoided as part of any diagnostic work-up as it has a poor sensitivity, specificity, and predictive value. In addition, the result remains positive for life, and immunoglobulin M (IgM) antibodies may occur during recurrent infections and are neither sensitive nor specific.^40,41^ However, blood for treponemal and RPR testing (or another nontreponemal test) should be collected at initial presentation of GUD to identify active syphilis and, if positive, the RPR may be used to monitor RPR titres as a marker of response to treatment. If treatment is not initially provided, a repeat RPR should be collected at the time of treatment to serve as the baseline titre. It is important to note that syphilis serological test (both RPR and treponemal tests) and the recently introduced RDTs may be negative in early primary syphilis (taking 1–4 weeks after the initial appearance of the chancre to become positive) and therefore a negative result does not exclude syphilis.^42^

## 4. Engaging sex partners in care

Treatment of STIs should include a discussion about safer sexual practices, emphasis on condom with lubricant use and, if HIV-uninfected, HIV PrEP should be offered.^16,17^ Efforts should be made to link to care the recent sex partners of patients with an STI. This is important for good sexual healthcare (as many STIs are asymptomatic), and for the prevention of re-infection. To avoid STI transmission to the patients’ sex partners, patients diagnosed with an STI should be advised to abstain from sex or to use condoms consistently for at least one week following completion of treatment.

Patients should be informed about the importance of partner treatment and provided with a notification slip for their sex partners to facilitate linkage to care. The notification slip should specify the syndrome that the index patient was treated for and, ideally, also specific pathogens that have been detected. This aids appropriate partner treatment at any healthcare facility, regardless of the presence of symptoms. Sex partners should receive the same antimicrobial treatment regimen as the index case (Table 3) but with two exceptions: men should be given a single dose (instead of a seven-day course) of metronidazole, and asymptomatic partners of a patient with GUD should be treated with BPG only and not be given acyclovir in addition.

Expedited partner therapy (EPT) for STIs is practised in several countries globally but is currently not supported by Southern African law, despite promising results in research studies.^23,43^ In EPT, index patients who have had an STI diagnosed are provided with a pill pack based on their laboratory test result that contains a single oral dose of azithromycin (for *C. trachomatis*) and/or cefixime (for *N. gonorrhoeae*) to give to their recent sex partners.

## 5. Clinical management of patients with recurrent or persistent symptoms

Symptom recurrence or persistence may occur for various reasons. Causes include reinfection from the same sex partner in the case of unsuccessful partner treatment, a repeat infection from a new sex partner, poor treatment compliance, symptom aetiology that is not covered by the initial empirical regimen, suboptimal treatment efficacy of medications for specific STIs, AMR and other non-infectious aetiologies (Table 4).^18,44^

**TABLE 4:** Reasons for persistent or recurrent sexually transmitted infection episodes.

Cause	Mechanisms
Sexual partner was not treated	Reinfection by same sex partner
Sexual partner has other partners	New infection with similar or different pathogen
New sexual partner	New infection
Poor treatment compliance	Persistent infection (treatment failure)
Aetiology not covered by initial treatment†	Persistent infection
Treatment with suboptimal efficacy‡	Persistent infection (treatment failure)
Vomiting after treatment	Persistent infection (treatment failure)
Antimicrobial resistance	Treatment failure
Other non-infectious aetiology	No infection

† , For example, *Trichomonas vaginalis* in men and vulvo-vaginal candidiasis in women.

‡ , For example, single dose of metronidazole for *Trichomonas vaginalis*.

Based on the available surveillance and research data, AMR to medications used in most STI first-line syndromic regimens is uncommon in Southern Africa. Nevertheless, AMR in *N. gonorrhoeae* and *M. genitalium* is on the rise globally and evaluation for AMR should be included in any diagnostic work-up.^24,45,46^

It is essential to take a thorough sexual and clinical history, and to conduct a physical examination of all patients presenting with persistent or recurrent MUDS or VDS. If reinfection, new infection, or poor treatment adherence is likely, then retreatment with the same empirical STI regimen is indicated. In the case of first-line treatment failure for MUDS, retreatment with ceftriaxone 1 g intramuscularly can be provided to overcome possible elevation in the minimum inhibitory concentration (MIC) of some *N. gonorrhoeae* strains. Should resources allow, a culture for AMR testing should be obtained. Increasing the ceftriaxone dose is not indicated for women as it results in a high level of overtreatment due to the low prevalence of *N. gonorrhoeae* in VDS. Women with persistent VDS who have received single-dose metronidazole as initial treatment should be given a 7-day oral course of metronidazole.

If the scenarios described have been excluded, then referral of patients with persistent or recurrent MUDS or VDS to an expert clinician with access to diagnostic testing is indicated to establish the aetiology of symptoms (including non-infectious causes) and to detect possible AMR. Similarly, individuals with persistent GUD should be referred to a centre with laboratory capacity and clinical expertise to diagnose the various STIs, skin and other differential diagnoses.

## 6. Diagnostic testing for sexually transmitted infections

Diagnostic testing can be used to screen asymptomatic individuals for STIs, to guide treatment and partner management of symptomatic individuals, and to inform management of complicated cases with persistent or recurrent symptoms (Tables 5a and 5b).

**TABLE 5a:** Recommended diagnostic tests for management of sexually transmitted infection-associated symptoms – Genital discharge syndromes.

Pathogen or syndrome	Management of first episode MUDS or VDS cases†	Management of persistent or recurrent cases‡
***Chlamydia trachomatis***	NAAT	NAAT
***Neisseria gonorrhoeae***	Urethral smear microscopy for presumptive diagnosis NAAT	NAAT Culture with AST
***Trichomonas vaginalis***	NAAT preferred Microscopy or antigen test valuable if positive result	NAAT
***Mycoplasma genitalium***	Not indicated	NAAT followed by test for azithromycin resistance when NAAT for NG/CT are negative
**Bacterial vaginosis**	Microscopy	Microscopy Multiplex NAATs (BV panel)
**Vulvo-vaginal candidiasis**	Microscopy	Microscopy Fungal culture with AST

MUDS, male urethral discharge syndrome; VDS, vaginal discharge syndrome; NAAT, nucleic acid amplification test; AST, antimicrobial susceptibility testing; BV, bacterial vaginosis; NG, Niesseria gonorrhoeae; CT, *Chlamydia Trachomatis*.

**TABLE 5b:** Recommended diagnostic tests for management of sexually transmitted infection-associated symptoms – Genitalulcer ulcer disease.

Pathogen or syndrome	Management of GUD cases	Management of persistent or recurrent GUD
**Herpes simplex virus**	NAAT of ulcer swab	NAAT of ulcer; with molecular resistance test if indicated
***Treponema pallidum* (syphilis)**	NAAT of ulcer swab Serology	NAAT of ulcer Serology
***Haemophilus ducreyi* (chancroid)**	Not indicated	NAAT of ulcer or lymph node aspirate
***Chlamydia trachomatis* biovar L1-L3 (LGV)**	Not indicated	NAAT of ulcer or lymph node aspirate

NAAT, nucleic acid amplification test; GUD, genital ulcer disease; LGV, lymphogranuloma venereum.

† , To provide same-day targeted treatment and/or to guide partner management.

‡ , To provide targeted antimicrobial treatment.

### 6.1. *Chlamydia trachomatis, Neisseria gonorrhoeae* and *Trichomonas vaginalis*

Currently, there are no point-of-care tests available for *C. trachomatis, N. gonorrhoeae and T. vaginalis* that meet the WHO target product profile. However, in-facility NAATs (such as GeneXpert^®^) are used for same-day diagnosis. On-site light microscopy using methylene blue- or Gram-stained urethral smears may be used by an experienced healthcare worker to establish a rapid presumptive diagnosis of *N. gonorrhoeae*.^47^ The Amsel criteria or light microscopy (using the Hay and Ison scoring system or Nugent score) of vaginal discharge smears may be used for diagnosis of BV, and VVC can be diagnosed through visualisation of yeasts or pseudohyphae.^15^ In addition, wet mount microscopy can confirm a diagnosis of *T. vaginalis*; however, the absence of visible motile protozoa does not rule out a diagnosis of trichomoniasis.^6^ Bacterial culture and drug susceptibility testing is only indicated when there are concerns about drug-resistant *N. gonorrhoeae* infection. Serology is not indicated.

Molecular detection using a high-quality NAAT is considered the standard of care for these STIs. There is a large variety of available high-quality molecular assays with good diagnostic performance. These can be manual or automated and either single or high throughput. For NAAT, first-catch urine for men and a self-collected vulvovaginal or healthcare-worker-collected endocervical swab for women is the preferred specimen. The acceptability and feasibility of self-collected vaginal swabs in women is high.^48,49,50^ Dependent on sexual practices, anorectal or pharyngeal swabs may be healthcare-worker- or self-collected in MSM, TGD and CSW. Molecular testing for the LGV biovar *C. trachomatis* is indicated in the case of rectal *C. trachomatis* infection and may be useful in the differential diagnosis of genital ulcers.

A test to confirm cure is not indicated in individuals with a urogenital infection that has been treated using the recommended antimicrobial regimen. An NAAT may remain positive for several weeks following completion of treatment as a result of the presence of non-viable organisms and uncleared DNA in the genital tract.^51,52^ Based on sexual practices, a repeat test at three or six months after the initial episode may be indicated (Table 1).

### 6.2. Mycoplasma genitalium

*M. genitalium* can only be detected by NAAT, and diagnostic testing is only recommended in patients with persistent or recurrent discharge and negative *N. gonorrhoeae* and *C. trachomatis* tests. Given the rapid global emergence of macrolide resistance over the past decade, it is recommended, if available, to combine testing for *M. genitalium* with macrolide resistance testing despite the relatively low prevalence of resistance in Southern Africa.

Molecular testing for *Mycoplasma hominis* and *Ureaplasma* species is included in some multiplex assays; however, these tests are not recommended as part of routine practice. Such testing should only be considered in specific populations as the pathogenicity of these bacteria is uncertain and the relevance of treatment unclear.

### 6.3. Herpes simplex virus-1 and herpes simplex virus-2

The diagnosis of HSV is mainly clinical. An NAAT of a swab of a genital lesion is the diagnostic standard for HSV-1 and HSV-2 infection. These NAAT assays have a sensitivity of > 90% and are considered highly specific.^15,37^ Molecular antiviral resistance testing is indicated in chronic ulcers not responding to prolonged acyclovir treatment, especially in PLHIV, and histology may also be indicated to aid in a differential diagnosis. Herpes simplex virus serology testing should not form part of STI management because of poor test sensitivity, specificity, and predictive values, antibody positivity that may last for life, and IgM antibodies that may occur during recurrent infection.^40,41^

### 6.4. Treponema pallidum

There is no single standard test for diagnosis of syphilis. In patients presenting with presumptive syphilis, molecular detection of *T. pallidum* from an ulcer swab may be used to confirm early diagnosis but, because of a test sensitivity of 80% – 90%, syphilis cannot be excluded by a negative test.^38,39^

Treponemal RDTs may be used to diagnose syphilis in asymptomatic individuals; however, these tests are unreliable when it comes to diagnosing primary syphilis due to the delay in antibody response and the limitation of antibody detection as compared to a laboratory assay. If RDTs are not available, laboratory testing using the so-called inverse algorithm can be used. This is when an automated specific treponemal antibody test (e.g. *T. pallidum* enzyme or chemiluminescent immunoassay) is used as the first step in diagnosis and, if positive, is followed by a reflex nontreponemal assay (e.g. RPR). Alternatively, in symptomatic individuals, the traditional algorithm could be used (i.e. a nontreponemal test which is quantitated to determine titre is then followed by a specific treponemal antibody test in the case of a positive test).

## 7. Pathogen-directed treatment of specific sexually transmitted infections

Unlike syndromic treatment that aims to cover the most likely aetiology, pathogen-directed treatment of STIs aims to achieve the highest possible cure rate using the most optimal antimicrobial therapy.

### 7.1. Chlamydia trachomatis

Treatment of *C. trachomatis* is usually covered with azithromycin in syndromic treatment regimens. However, a recent meta-analysis has shown higher treatment efficacy (lower rate of microbiological failure but not clinical failure) with a 7-day course of doxycycline for urogenital infection in men.^53^ In addition, recent trials have shown a higher efficacy of doxycycline as compared to azithromycin for the treatment of rectal *C. trachomatis* infection in MSM and women.^54,55^ Concurrent treatment of rectal infection in women is important as auto-inoculation of cervical infection may occur resulting in persistent infection. In addition, rectal infections may remain untreated in MSM and women posing an increased risk for HIV transmission.^56,57^ Therefore, doxycycline is recommended as the most effective directed first-line treatment for *C. trachomatis* (Table 6). Azithromycin provides a good alternative when managing symptomatic individuals with MUDS or VDS, when there are concerns about tolerability and when adherence may pose a problem in completion of the 7-day course of doxycycline.

**TABLE 6:** Recommended antimicrobial drugs for targeted treatment of uncomplicated sexually transmitted infections.

Pathogen	First-line option	Effective substitutes
*Chlamydia trachomatis*	Doxycycline 100 mg orally, 2 times per day for 7 days	Azithromycin 1 g orally, single dose
*Neisseria gonorrhoeae*	Ceftriaxone 500 mg single intramuscular injection†	Cefixime 800 mg, orally, single dose
*Trichomonas vaginalis* (women)	Metronidazole 400 mg/500 mg, orally, 2 times per day for 7 days‡	Metronidazole 2 g, orally, single dose *OR* Tinidazole, orally, 2 g single dose
*Trichomonas vaginalis* (men)	Metronidazole 2 g, orally, single dose	Metronidazole 400 mg/500 mg, orally, 2 times per day for 7 days‡
*Mycoplasma genitalium*	Doxycycline 100 mg, orally, two times per day for 7 days followed by: Azithromycin 1 g initial dose followed by 500 mg, orally, daily for 3 additional days if unknown resistance profile or macrolide-susceptible Moxifloxacin, orally, 400 mg daily for 7 days if macrolide-resistant	To discuss with specialist
**Herpes simplex**	Primary infection: Acyclovir 400 mg, orally, 3 times per day for up to 10 days Recurrent infection: Acyclovir 400 mg, orally, 3 times a day for 5 days OR 800 mg 3 times a day for 2 days	Primary infection: Valaciclovir 500 mg, orally, twice daily for up to 10 days Recurrent ulcer: Valaciclovir 500 mg, orally, twice daily for 3 days
***Treponema pallidum* (syphilis)**	Early syphilis§: Benzathine benzylpenicillin 2.4 million units, intramuscularly, single dose Late syphilis: Benzathine benzylpenicillin 2.4 million units, intramuscularly, single dose, once weekly for three consecutive weeks	Early syphilis§: Doxycycline 100 mg, orally, twice per day for 14 days OR Late syphilis: Procaine penicillin 1.2 million units intramuscular injection once daily for 20 consecutive days OR Doxycycline 100 mg, orally, twice per day for 30 days

† , lncrease dose to 1 g intramuscular injection in case of confirmed oropharyngeal infection.

‡ , 400 mg or 500 mg based on local availability.

§ , Early syphilis: primary, secondary, or early latent (< 2 years ago); late syphilis does not include management of neurosyphilis.

### 7.2. Neisseria gonorrhoeae

*N. gonorrhoeae* has been treated for the past decade using combination therapy with prevention of resistance as an important focus. However, such an effect has not been documented and the decreasing rate of azithromycin susceptibility of *N. gonorrhoeae* and reported treatment failures of oropharyngeal gonorrhoea are an important concern. Some international guidelines, based on local susceptibility data,^14,58,59^ recommend a single dose of 500 mg or 1 g of ceftriaxone for documented *N. gonorrhoeae* infection. Based on the locally available susceptibility data this guideline recommends a 500 mg intramuscular injection of ceftriaxone without the addition of azithromycin for the treatment of confirmed genital *N. gonorrhoeae*. The ceftriaxone dose should be increased to 1 g in the case of confirmed oropharyngeal infection due to the lower bioavailability of ceftriaxone in the oropharynx. Ceftriaxone as an intramuscular injection, or cefixime 800 mg given as an oral dose, is preferred over intramuscular gentamicin as it has a higher efficacy for both uncomplicated urogenital and extragenital infections.^60,61^

Ceftriaxone-resistance in *N. gonorrhoeae* is a category 3 notifiable condition in South Africa and many other countries. Should such resistance be identified, treatment should be based on the full phenotypic and genotypic drug susceptibility profile and guided by expert clinical advice.

### 7.3. Trichomonas vaginalis

Metronidazole is the mainstay of *T. vaginalis* treatment. A 7-day oral course of 400 mg or 500 mg twice daily has a substantially higher efficacy than a 2 g single dose, regardless of the patient’s HIV-infection status. However, persistence may still occur in approximately 10% of women.^33^ Tinidazole is a good alternative to a course of metronidazole in women with persistent infection. There are currently no randomised controlled trial data for *T. vaginalis* treatment in men, but single-dose metronidazole treatment has reportedly high cure rates.

### 7.4. Mycoplasma genitalium

A 1 g single oral dose of azithromycin used in syndromic management may treat macrolide susceptible *M. genitalium* infection but may also facilitate the emergence of macrolide resistance in this pathogen. Macrolide resistance has emerged globally; however, limited data for the African region (including South Africa and Kenya) suggest that local macrolide resistance levels are still low.^24,46,56,59,62^ In the absence of resistance testing and given the widespread use of moxifloxacin (an alternative treatment) in management of tuberculosis, a course of azithromycin is still recommended as first-line treatment of *M. genitalium* (Table 6). Resistance-guided therapy is preferred in settings where macrolide resistance testing is available. This two-stage therapy consists of treatment with doxycycline to reduce organism load and facilitate clearance, followed by treatment with azithromycin or moxifloxacin based on the resistance test result.^15^

### 7.5. Herpes simplex virus-1 and Herpes simplex virus-2

HSV-1 and HSV-2 ulcers should be treatment with acyclovir or valacyclovir. Alternative treatment may be given based on the result of acyclovir resistance testing in cases of persistent ulceration unresponsive to standard acyclovir therapy. In general, based on the limited data from surveillance, acyclovir resistance in HSV is uncommon in our region.^63^

### 7.6. Treponema pallidum

Benzathine benzylpenicillin, or BPG, is the mainstay of syphilis treatment with high cure rates and no resistance documented. Single injections are sufficient for patients with manifestations of primary, secondary, or early latent syphilis infection. However, a series of three injections is recommended for all asymptomatic individuals with a syphilis diagnosis and no documented negative syphilis serology in the past year. Asymptomatic individuals with a documented negative syphilis treatment serology in the past two years may be treated with a single BPG injection. In case of suspected or confirmed penicillin allergy, penicillin-desensitisation is recommended and is preferred over an alternative treatment.

Global shortages of benzathine benzylpenicillin have accelerated the search for alternative treatment regimens. If BPG is unavailable, doxycycline may be given for primary, secondary or latent syphilis in adult men and non-pregnant women. The efficacy of amoxicillin and oral cephalosporins is currently being studied. Resistance to macrolides in *T. pallidum* is common globally and these medications should be avoided in the treatment of syphilis. Management of tertiary syphilis (e.g. neurosyphilis) is beyond the scope of this guideline and should be managed in a specialist setting.

## 8. Conclusion

Symptom screening and syndromic management have been the cornerstone of STI control for the past decades in Southern Africa; however, the burden of STIs remains high.^1,45^ Strengthening STI screening, diagnosis and case management is essential to improve health outcomes and to control the STI epidemic. If resources are available, diagnostic screening and testing, combined with pathogen-directed treatment of STIs is essential to improve patient outcomes and make significant progress towards STI control, while mitigating the effects of rising levels of AMR. In addition, the development and implementation of RDTs for the most common STIs is urgently required for the strengthening of STI case management.^64^ Strengthening STI care in Southern Africa requires the empowerment of patients, capacity building of healthcare workers, and investment in diagnostic and therapeutic resources and infrastructure. This guideline provides an important step towards evidence-based, best-practice and effective STI management in Southern Africa.
