# Erratum: Southern African HIV Clinicians Society 2022 guideline for the management of sexually transmitted infections: Moving towards best practice

**DOI:** 10.4102/sajhivmed.v23i1.1465

**Published:** 2022-11-24

**Authors:** Remco P.H. Peters, Nigel Garrett, Nomathemba Chandiwana, Ranmini Kularatne, Adrian J. Brink, Karen Cohen, Katherine Gill, Thato Chidarikire, Camilla Wattrus, Jeremy S. Nel, Mahomed Y.S. Moosa, Linda-Gail Bekker

**Affiliations:** 1 Research Unit, Foundation for Professional Development, East London, South Africa; 2 Department of Medical Microbiology, University of Pretoria, Pretoria, South Africa; 3 Division of Medical Microbiology, Faculty of Health Sciences, University of Cape Town, Cape Town, South Africa; 4 Centre for the AIDS Programme of Research in South Africa (CAPRISA), University of KwaZulu-Natal, Durban, South Africa; 5 Department of Public Health Medicine, School of Nursing and Public Health, University of KwaZulu-Natal, Durban, South Africa; 6 Ezintsha, Faculty of Health Science, University of the Witwatersrand, Johannesburg, South Africa; 7 Department of Clinical Microbiology and Infectious Diseases, Faculty of Health Sciences, University of the Witwatersrand, Johannesburg, South Africa; 8 Department of Medicine, Division of Clinical Pharmacology, University of Cape Town, Cape Town, South Africa; 9 Desmond Tutu HIV Centre, University of Cape Town, Cape Town, South Africa; 10 National Department of Health, Pretoria, South Africa; 11 Southern African HIV Clinicians Society (SAHCS), Johannesburg, South Africa; 12 Helen Joseph Hospital, University of the Witwatersrand, Johannesburg, South Africa; 13 Department of Infectious Disease, Division of Internal Medicine, Nelson R. Mandela School of Medicine, University of KwaZulu-Natal, Durban, South Africa

In the published article, Peters RPH, Garrett N, Chandiwana N, et al. Southern African HIV Clinicians Society 2022 guideline for the management of sexually transmitted infections: Moving towards best practice. S Afr J HIV Med. 2022;23(1):a1450. https://doi.org/10.4102/sajhivmed.v23i1.1450, a typographical error occurred, where HSV-1 was used instead of HSV-2. The correction has now been made on page 6, in Section 3. Clinical management of the symptomatic patient, 3.3. Genital ulcer disease, paragraph one, and should read:

The original paragraph:

The manifestation of GUD is diverse and the characteristics of the ulcer (e.g. presence or absence of pain, shape of edges, multiplicity) are of poor diagnostic value in determining aetiology, particularly in PLHIV.^34^ Attempting to clinically diagnose the aetiology of GUD using ulcer characteristics is not recommended and should not be used to inform treatment decisions. HSV-1 and HSV-1 are the most common causes of genital ulcers followed by *Treponema pallidum*, the causative agent of syphilis. Lymphogranuloma venereum (LGV) caused by *C. trachomatis* biovars L1–L3, chancroid (*Haemophilus ducreyi*), and donovanosis (*Klebsiella granulomatis*) have become uncommon in the last decade.^14,35,36^

The revised and updated paragraph:

The manifestation of GUD is diverse and the characteristics of the ulcer (e.g. presence or absence of pain, shape of edges, multiplicity) are of poor diagnostic value in determining aetiology, particularly in PLHIV.^34^ Attempting to clinically diagnose the aetiology of GUD using ulcer characteristics is not recommended and should not be used to inform treatment decisions. HSV-2 and HSV-1 are the most common causes of genital ulcers followed by *Treponema pallidum*, the causative agent of syphilis. Lymphogranuloma venereum (LGV) caused by *C. trachomatis* biovars L1–L3, chancroid (*Haemophilus ducreyi*), and donovanosis (*Klebsiella granulomatis*) have become uncommon in the last decade.^14,35,36^

The publisher apologises for this error. The correction does not change the study’s findings of significance or overall interpretation of the study’s results or the scientific conclusions of the article in any way.

